# Supplementary material for: Loss and Recovery of Genetic Diversity in Adapting Populations of HIV
Source: PLoS Genet. 2014 Jan 23;10(1):e1004000. doi: 10.1371/journal.pgen.1004000 (PMC3900388; doi:10.1371/journal.pgen.1004000)
Supplement: Figure S1 — Nucleotide diversity reduction due to different fixations. Nucleotide diversity at all sites. The patients are split in four groups: the first group in which one codon of the K103N was fixed (K103N one codon), a group in which a mixture of two codons of K103N was fixed (K103N two codons), a group in which a different amino acid change was fixed (Other mutation) and a group in which two resistance mutations were fixed (Two mutations). Large red circles show the median for each group. The shape of the points reflects the treatment the patient was on: circle: treated with indinavir then later switched to efavirenz combination therapy, triangle: treated with indinavir + efavirenz, plus-sign: treated with indinavir, cross: treated with ZDV/3TC + efavirenz diamond: treated with ZDV/3TC, later switched to efavirenz combination therapy. (PDF) [file pgen.1004000.s001.pdf]

# Supplementary Figure S1

Loss and Recovery of Genetic Diversity in Adapting Populations of HIV
Pleuni S. Pennings , Sergey Kryazhimskiy , John Wakeley (PLoS Genetics)

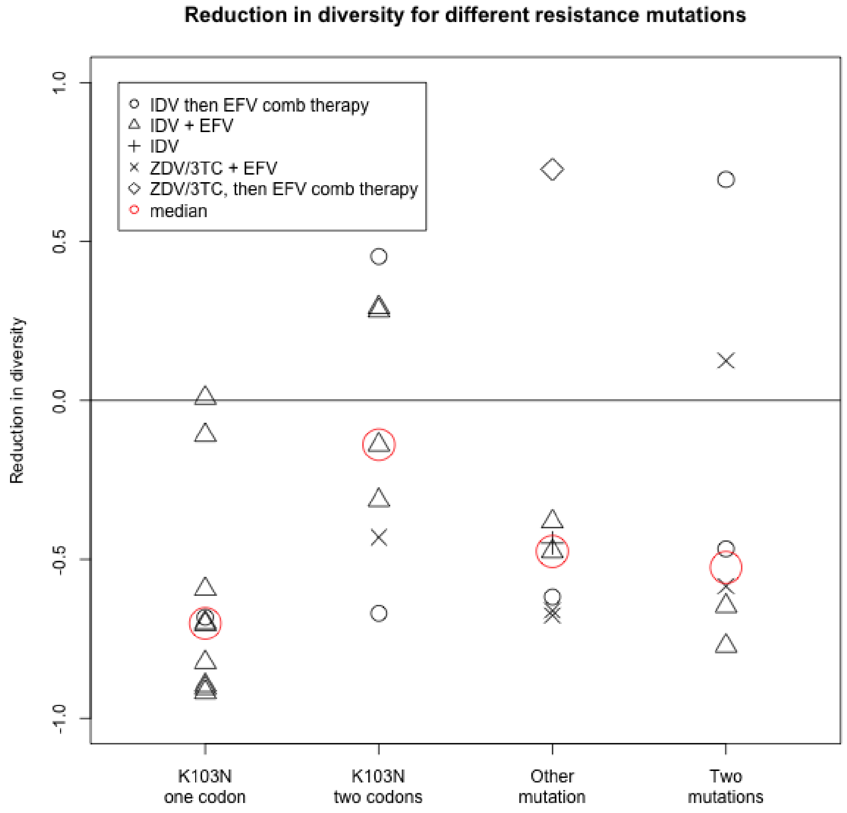


**Supplementary Figure S1. Nucleotide diversity reduction due to different fixations.** Nucleotide diversity at all sites. The patients are split in four groups: the first group in which one codon of the K103N was fixed (K103N one codon), a group in which a mixture of two codons of K103N was fixed (K103N two codons), a group in which a different amino acid change was fixed (Other mutation) and a group in which two resistance mutations were fixed (Two mutations). Large red circles show the median for each group. The shape of the points reflects the treatment the patient was on: circle: treated with indinavir then later switched to efavirenz combination therapy, triangle: treated with indinavir + efavirenz, plus-sign: treated with indinavir, cross: treated with ZDV/3TC + efavirenz diamond: treated with ZDV/3TC, later switched to efavirenz combination therapy.
