# Supplementary material for: Loss and Recovery of Genetic Diversity in Adapting Populations of HIV
Source: PLoS Genet. 2014 Jan 23;10(1):e1004000. doi: 10.1371/journal.pgen.1004000 (PMC3900388; doi:10.1371/journal.pgen.1004000)
Supplement: Figure S2 — Soft and hard sweep example. A. Soft sweep in patient 089. Codon GGC coding for glysine at position 190 was replaced by day 56 by codon AGC coding for serine, which confers resistance to NNRTI drugs. The G190S mutation appears to have occurred on at least two different backgrounds. As predicted by theory [18], the site is embedded in a region of high linkage disequilibrium. The different haplotypic backgrounds are indicated by differently colored pastel backgrounds. Genetic diversity was reduced by . The plot shows the polymorphic sites in the reverse transcriptase region, excluding all singletons. Each row represents a sequenced viral isolate. Each column represents a polymorphic site, with the derived synonymous and non-synonymous polymorphisms shown in black and orange respectively. Codons 103, 108, 188, 190 and 225 are all linked to NNRTI resistance and are shown explicitly. They are grey when when in the susceptible state and blue when in the resistant state. Mutations in these codons are colored pink. B. Hard sweep in patient 159. Codon GGA coding for glysine at position 190 was replaced by day 225 by codon GCA coding for alanine, which confers resistance to NNRTI drugs. The G190A mutation appears to have occurred on at least a single haplotypic background. Genetic diversity was reduced by . The plot shows the polymorphic sites in the reverse transcriptase region, excluding all singletons. Each row represents a sequenced viral isolate. Each column represents a polymorphic site, with the derived synonymous and non-synonymous polymorphisms shown in black and orange respectively. Codons 190 is shown explicitly. It is colored grey when when in the susceptible state and blue when in the resistant state. Mutations in this codon are colored pink. (PDF) [file pgen.1004000.s002.pdf]

Supplementary Figure S2

Loss and Recovery of Genetic Diversity in Adapting Populations of HIV
Pleuni S. Pennings , Sergey Kryazhimskiy , John Wakeley (PLoS Genetics)

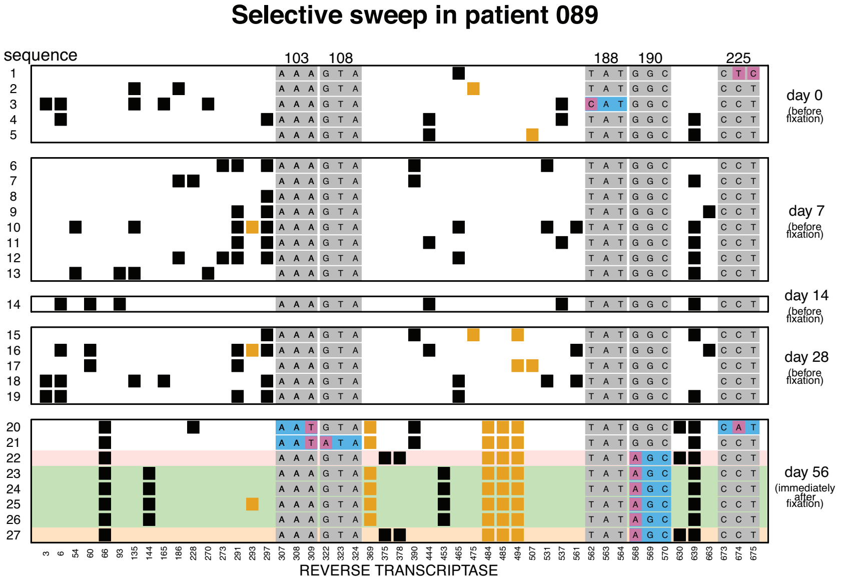

Supplementary Figure S2. Soft and hard sweep example. A. Soft sweep in patient 089. Codon GGC coding for glysine at position 190 was replaced by day 56 by codon AGC coding for serine, which confers resistance to NNRTI drugs. The G190S mutation appears to have occurred on at least two different backgrounds. As predicted by theory [1], the site is embedded in a region of high linkage disequilibrium. The different haplotypic backgrounds are indicated by differently colored pastel backgrounds. Genetic diversity was reduced by 38%. The plot shows the polymorphic sites in the reverse transcriptase region, excluding all singletons. Each row represents a sequenced viral isolate. Each column represents a polymorphic site, with the derived synonymous and non-synonymous polymorphisms shown in black and orange respectively. Codons 103, 108, 188, 190 and 225 are all linked to NNRTI resistance and are shown explicitly.

References

1. Pennings P, Hermisson J (2006) Soft sweeps III: The signature of positive selection from recurrent mutation. PLoS Genetics 2: e186.

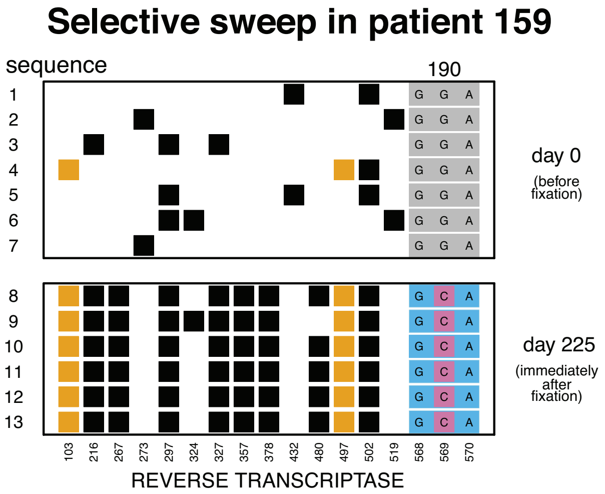

Supplementary Figure S2. Soft and hard sweep example. B. Hard sweep in patient 159. Codon GGA coding for glysine at position 190 was replaced by day 225 by codon GCA coding for alanine, which confers resistance to NNRTI drugs. The G190A mutation appears to have occurred on at least a single haplotypic background. Genetic diversity was reduced by 67%. The plot shows the polymorphic sites in the reverse transcriptase region, excluding all singletons. Each row represents a sequenced viral isolate. Each column represents a polymorphic site, with the derived synonymous and non-synonymous polymorphisms shown in black and orange respectively. Codons 190 is shown explicitly.
