# Supplementary material for: Loss and Recovery of Genetic Diversity in Adapting Populations of HIV
Source: PLoS Genet. 2014 Jan 23;10(1):e1004000. doi: 10.1371/journal.pgen.1004000 (PMC3900388; doi:10.1371/journal.pgen.1004000)
Supplement: Figure S3 — Soft and hard sweep example. A. Soft sweep in patient 6 [20] Codon ATG coding for methionine at position 46 was replaced in 1993/10 by codons CTG and TTG, both coding for leucine, which confers resistance to Protease inhibitor drugs. The plot shows the polymorphic sites in the protease region, excluding all singletons. Each row represents a sequenced viral isolate. Each column represents a polymorphic site, with the derived synonymous and non-synonymous polymorphisms shown in black and orange respectively. Codons 10, 46, 71 are linked to Protease Inhibitor drug resistance and shown explicitly. They are colored grey when when in the susceptible state and blue when in the resistant state. Mutations in these codons are colored pink. The viral load in this patient was around before treatment, it went down approximately log values and rebounded quickly to its original level (see figure 1 in [20]). B. Hard sweep in patient 3 [20] Codon ATG coding for methionine at position 46 was replaced in 1994/02 by codon TTG, which codes for leucine, which confers resistance to Protease inhibitor drugs. The plot shows the polymorphic sites in the protease region, excluding all singletons. Each row represents a sequenced viral isolate. Each column represents a polymorphic site, with the derived synonymous and non-synonymous polymorphisms shown in black and orange respectively. Codons 10, 46, 71 are linked to Protease Inhibitor drug resistance and shown explicitly. They are colored grey when when in the susceptible state and blue when in the resistant state. Mutations in these codons are colored pink. The viral load in this patient was slightly higher than , it went down to below the detection limit of and stayed low for more than six months, after which it rebounded (see figure 1 in [20]). (PDF) [file pgen.1004000.s003.pdf]

Supplementary Figure S3

Loss and Recovery of Genetic Diversity in Adapting Populations of HIV
Pleuni S. Pennings , Sergey Kryazhimskiy , John Wakeley (PLoS Genetics)

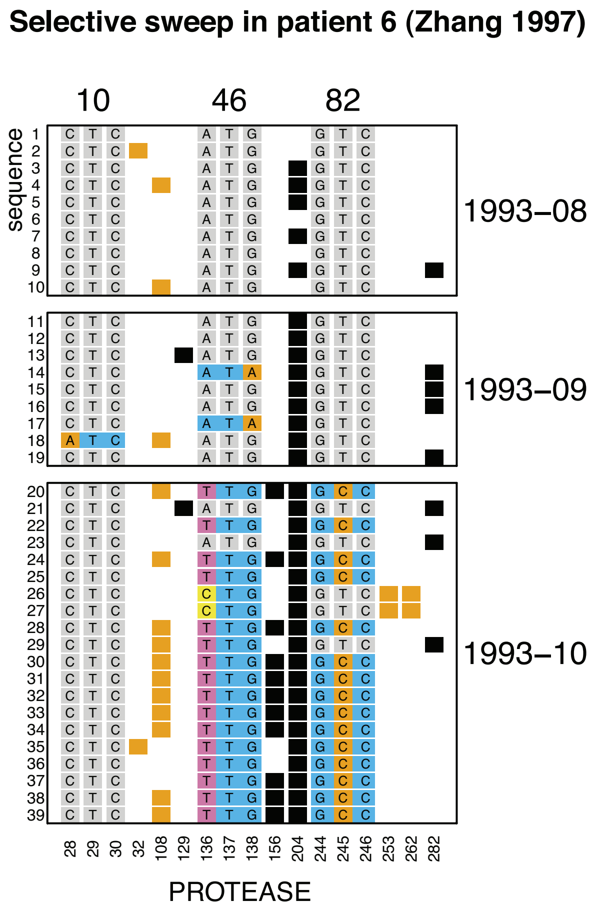

Supplementary Figure S3. Soft and hard sweep example. A. Soft sweep in patient 6 [1] Codon ATG coding for methionine at position 46 was replaced in 1993/10 by codons CTG and TTG, both coding for leucine, which confers resistance to Protease inhibitor drugs. The plot shows the polymorphic sites in the protease region, excluding all singletons. Each row represents a sequenced viral isolate. Each column represents a polymorphic site, with the derived synonymous and non-synonymous polymorphisms shown in black and orange respectively. Codons 10, 46, 71 are linked to Protease Inhibitor drug resistance and shown explicitly. The viral load in this patient was around 10^6 before treatment, it went down approximately 1.5 log values and rebounded quickly to its original level (see figure 1 in [1]).

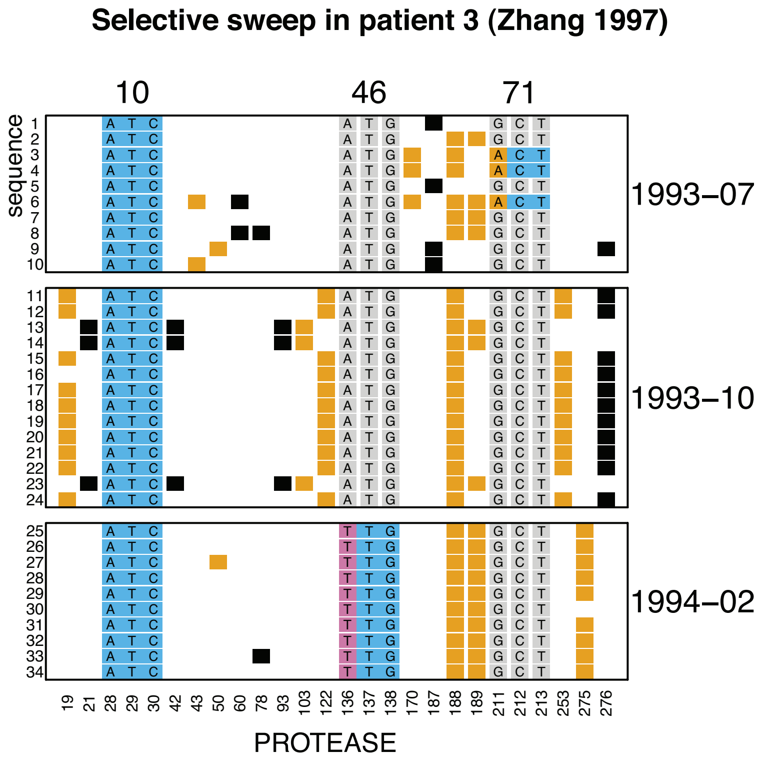

Supplementary Figure S3. Soft and hard sweep example.
B. Hard sweep in patient 3 [1] Codon ATG coding for methionine at position 46 was replaced in 1994/02 by codon TTG, which codes for leucine, which confers resistance to Protease inhibitor drugs. The plot shows the polymorphic sites in the protease region, excluding all singletons. Each row represents a sequenced viral isolate. Each column represents a polymorphic site, with the derived synonymous and non-synonymous polymorphisms shown in black and orange respectively. Codons 10, 46, 71 are linked to Protease Inhibitor drug resistance and shown explicitly. The viral load in this patient was slightly higher than 10^5, it went down to below the detection limit of 10^4 and stayed low for more than six months, after which it rebounded (see figure 1 in [1]).

References

1. Zhang Y, Imamichi H, Imamichi T, Lane C, J F, et al. (1997) Drug resistance during indinavir therapy is caused by mutations in the protease gene and in its gag substrate cleavage sites. Journal of Virology 71: 6662–6670.
