# Supplementary material for: Loss and Recovery of Genetic Diversity in Adapting Populations of HIV
Source: PLoS Genet. 2014 Jan 23;10(1):e1004000. doi: 10.1371/journal.pgen.1004000 (PMC3900388; doi:10.1371/journal.pgen.1004000)
Supplement: Figure S4 — Probability of a single origin (hard sweep) in a sample of size 6. Simulation results show that the number of origins is independent of whether or not standing genetic variation is involved, and if standing genetic variation is involved, the number of origins is independent of the selection coefficient of the allele before or after the start of treatment. The horizontal line denotes the theoretical expectation [4]. The bars denote the probability that a sample of size six consists of just one origin, averaged over 1000 runs. The population size in the simulations is and the mutation rate , so that . (PDF) [file pgen.1004000.s004.pdf]

Supplementary Figure S4

Loss and Recovery of Genetic Diversity in Adapting Populations of HIV
Pleuni S. Pennings , Sergey Kryazhimskiy , John Wakeley (PLoS Genetics)

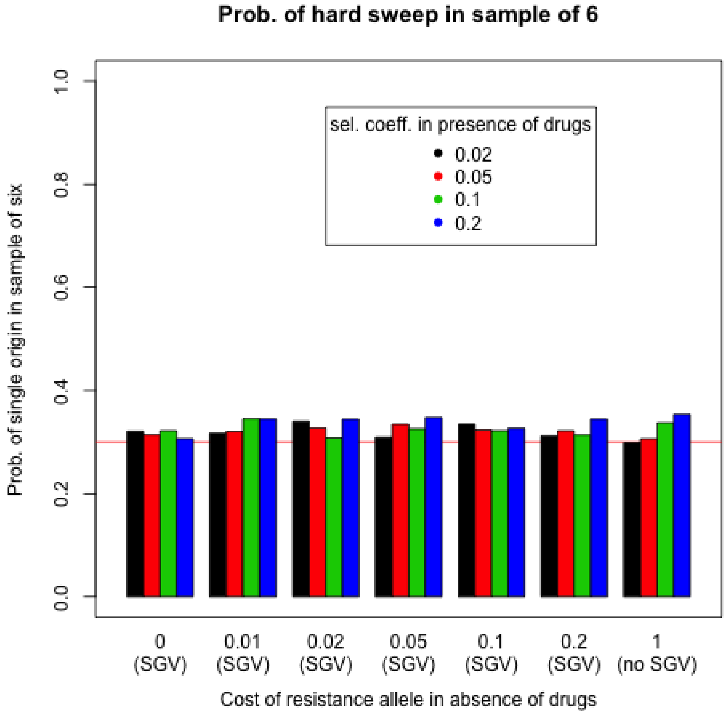

Supplementary Figure S4. Probability of a single origin (hard sweep) in a sample of size 6. Simulation results show that the number of origins is independent of whether or not standing genetic variation is involved, and if standing genetic variation is involved, the number of origins is independent of the selection coefficient of the allele before or after the start of treatment. The horizontal line denotes the theoretical expectation [1]. The bars denote the probability that a sample of size six consists of just one origin, averaged over 1000 runs. The population size in the simulations is 150, 000 and the mutation rate 2×10^{-6}, so that $\theta/2 = 0.3$.

References

1. Pennings P, Hermisson J (2006) Soft sweeps II: Molecular population genetics of adaptation from recurrent mutation or migration. Molecular Biology and Evolution 23: 1076–1084.
