# Supplementary material for: Loss and Recovery of Genetic Diversity in Adapting Populations of HIV
Source: PLoS Genet. 2014 Jan 23;10(1):e1004000. doi: 10.1371/journal.pgen.1004000 (PMC3900388; doi:10.1371/journal.pgen.1004000)
Supplement: Figure S5 — Predicted response time. Comparison of the response time , defined as the time it takes for to recover of its loss of diversity, from the heuristic model and from simulations. Dashed lines show (for ) or (for ). Dots display averages over 1000 simulations of a haploid Wright-Fisher population with individuals for each pair of values of and . In all cases, . (PDF) [file pgen.1004000.s005.pdf]

Supplementary Figure S5

Loss and Recovery of Genetic Diversity in Adapting Populations of HIV
Pleuni S. Pennings , Sergey Kryazhimskiy , John Wakeley (PLoS Genetics)

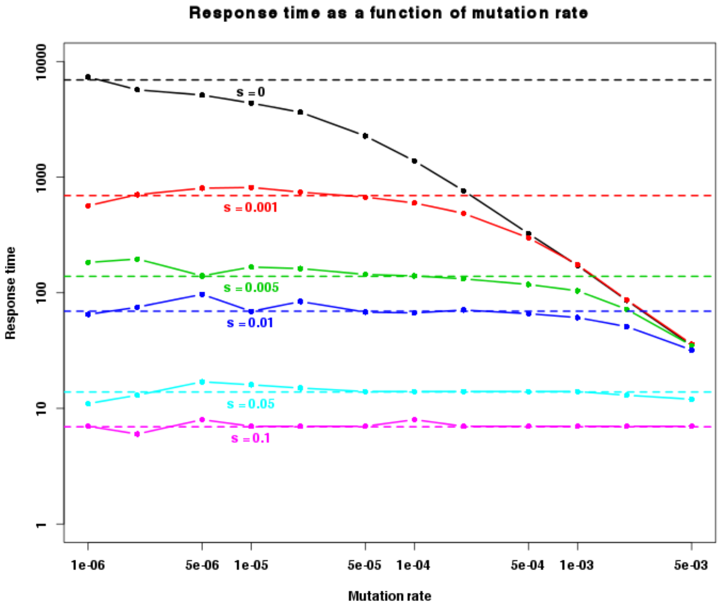

Supplementary Figure S5. Predicted response time. Comparison of the response time t_{half}, defined as the time it takes for H to recover 50% of its loss of diversity, from the heuristic model and from simulations. Dashed lines show $t_{half} = s^{-1}\log(2)$ (for $s > 0$) or $t_{half} = N\log(2)$ (for $s = 0$). Dots display averages over 1000 simulations of a haploid Wright-Fisher population with $N = 10^4$ individuals for each pair of values of u and s. In all cases, $v = u$.
