# Supplementary material for: Loss and Recovery of Genetic Diversity in Adapting Populations of HIV
Source: PLoS Genet. 2014 Jan 23;10(1):e1004000. doi: 10.1371/journal.pgen.1004000 (PMC3900388; doi:10.1371/journal.pgen.1004000)
Supplement: Table S1 — Patient details. For each of the (a.) 30 patients that were included in the analysis, this table gives (b.) the codon(s) of the resistance mutation(s) that occurred (with nucleotide changes indicated for all mutations except the 103rd codon of RT, P82 indicates the 82nd codon in Protease, all other codons are in RT), (c.) the level of diversity before and (d.) after the sweep, (e.) the sample size of the first sample after the sweep, (f.) the number of AAC and (g.) AAT alleles observed in the first sample after the sweep, and (h.) the treatment the patient was receiving. (PDF) [file pgen.1004000.s006.pdf]

Supplementary Table S1.

Loss and Recovery of Genetic Diversity in Adapting Populations of HIV
Pleuni S. Pennings , Sergey Kryazhimskiy , John Wakeley (PLoS Genetics)

Supplementary Table S1. Patient details. For each of the (a.) 30 patients that were included in the analysis, this table gives (b.) the codon(s) of the resistance mutation(s) that occurred (with nucleotide changes indicated for all mutations except the 103rd codon of RT, P82 indicates the 82nd codon in Protease, all other codons are in RT), (c.) the level of diversity before and (d.) after the sweep, (e.) the sample size of the first sample after the sweep, (f.) the number of AAC and (g.) AAT alleles observed in the first sample after the sweep, and (h.) the treatment the patient was receiving.

a. patient	b. resistance codon / mutation	c. div pre	d. div after	e. sam. size	f. 103 AAC	g. 103 AAT	h. treatment
5	103	0.018	0.006	4	3	1	IDV then EFV comb therapy
7	103	0.008	0.008	6	6	0	IDV + EFV
12	103	0.024	0.016	2	1	1	IDV + EFV
13	103	0.018	0.002	7	0	7	IDV + EFV
20	P82 (GTC → ACC)	0.017	0.010	6	0	0	IDV
22	103, P82 (GTC → GCC)	0.022	0.005	4	4	0	IDV + EFV
24	103	0.012	0.017	7	5	2	IDV then EFV comb therapy
32	103	0.012	0.004	8	8	0	IDV + EFV
44	103	0.019	0.017	3	3	0	IDV + EFV
56	103, 62 (GGC→ GTC)	0.012	0.004	2	2	0	IDV + EFV
57	103	0.010	0.013	6	3	3	IDV + EFV
58	103	0.012	0.016	7	4	3	IDV + EFV
63	103	0.012	0.005	7	7	0	IDV + EFV
66	103	0.016	0.003	5	5	0	IDV + EFV
70	P82 (GTC → GCC)	0.017	0.007	6	0	0	IDV then EFV comb therapy
71	103	0.018	0.005	7	0	7	IDV + EFV
77	103	0.025	0.002	7	0	7	IDV + EFV
81	103	0.012	0.010	8	7	1	IDV + EFV
83	190 (GGA → GCA)	0.015	0.008	8	0	0	IDV + EFV
86	103	0.029	0.003	6	0	6	IDV + EFV
87	103, P82 (GTC → GCC)	0.011	0.006	8	7	1	IDV then EFV comb therapy
89	190 (GGA → AGC)	0.020	0.012	8	0	0	IDV + EFV
91	103, P82 (GTC → GCC)	0.012	0.020	2	2	0	IDV then EFV comb therapy
95	103	0.012	0.004	6	6	0	IDV then EFV comb therapy
154	103, 100 (TTA → ATA)	0.020	0.008	7	7	0	ZDV/3TC + EFV
159	190 (GGA → GCA)	0.015	0.005	6	0	0	ZDV/3TC + EFV
166	103, 184 (ATG → GTG)	0.009	0.011	4	4	0	ZDV/3TC + EFV
167	184 (ATG → GTG)	0.015	0.026	7	0	0	ZDV/3TC, then EFV comb therapy
168	103	0.024	0.014	6	2	3	ZDV/3TC + EFV
171	184 (ATG → GTG)	0.024	0.008	2	0	0	ZDV/3TC + EFV
