# Supplementary material for: Loss and Recovery of Genetic Diversity in Adapting Populations of HIV
Source: PLoS Genet. 2014 Jan 23;10(1):e1004000. doi: 10.1371/journal.pgen.1004000 (PMC3900388; doi:10.1371/journal.pgen.1004000)
Supplement: Table S2 — List of amino acid changes that can be achieved by two or more single nucleotide changes and thus allow the observation of soft sweeps at the beneficial site itself. In almost all cases, the relevant mutations are transversions (TV). In the last three cases (marked with an asterisk), one of the mutations is a transition (TS). In these cases, the difference in mutation rates (higher for transitions) will make it less likely to observe a soft sweep. (PDF) [file pgen.1004000.s007.pdf]

Supplementary Table S2.

Loss and Recovery of Genetic Diversity in Adapting Populations of HIV
Pleuni S. Pennings , Sergey Kryazhimskiy , John Wakeley (PLoS Genetics)

Supplementary Table S2. List of amino acid changes that can be achieved by two or more single nucleotide changes and thus allow the observation of soft sweeps at the beneficial site itself. In almost all cases, the relevant mutations are transversions (TV). In the last three cases (marked with an asterisk), one of the mutations is a transition (TS). In these cases, the difference in mutation rates (higher for transitions) will make it less likely to observe a soft sweep.

a. From amino acid	b. From codon	c. To amino acid	d. To codons	e. Relevance for HIV drug resistance
K	AAA	N	AAC (TV), AAT (TV)	relevant for RT K103N
M	ATG	L	CTG (TV), TTG (TV)	relevant for RT M41L and PRO M46L
C	TGC	S	AGC (TV), TCC (TV)	
C	TGT	S	AGT (TV), TCT (TV)	
D	GAC	E	GAA (TV), GAG (TV)	
D	GAT	E	GAA (TV), GAG (TV)	
E	GAA	D	GAC (TV), GAT (TV)	
E	GAG	D	GAC (TV), GAT (TV)	
G	GGA	R	AGA (TS), CGA (TV)	
G	GGG	R	AGG (TS), CGG (TV)	
H	CAC	Q	CAA (TV), CAG (TV)	
H	CAT	Q	CAA (TV), CAG (TV)	
I	ATA	L	CTA (TV), TTA (TV)	
K	AAG	N	AAC (TV), AAT (TV)	
L	TTA	F	TTC (TV), TTT (TV)	
L	TTG	F	TTC (TV), TTT (TV)	
N	AAC	K	AAA (TV), AAG (TV)	
N	AAT	K	AAA (TV), AAG (TV)	
Q	CAA	H	CAC (TV), CAT (TV)	
Q	CAG	H	CAC (TV), CAT (TV)	
R	AGA	S	AGC (TV), AGT (TV)	
R	AGG	S	AGC (TV), AGT (TV)	
T	ACC	S	TCC (TV), AGC (TV)	
T	ACT	S	TCT (TV), AGT (TV)	
V	GTA	L	CTA (TV), TTA (TV)	
V	GTG	L	CTG (TV), TTG (TV)	
W	TGG	C	TGC (TV), TGT (TV)	
W	TGG	R	AGG (TV), CGG (TS)	
S	AGC	R	CGC (TV), AGA (TV), AGG (TV)	
S	AGT	R	CGT (TV), AGA (TV), AGG (TV)	
F	TTC	L	CTC (TS), TTA (TV), TTG (TV)	*
F	TTT	L	CTT (TS), TTA (TV), TTG (TV)	*
M	ATG	I	ATA (TS), ATC (TV), ATT (TV)	*
