# Supplementary material for: Loss and Recovery of Genetic Diversity in Adapting Populations of HIV
Source: PLoS Genet. 2014 Jan 23;10(1):e1004000. doi: 10.1371/journal.pgen.1004000 (PMC3900388; doi:10.1371/journal.pgen.1004000)
Supplement: Text S1 — Recovery of diversity. In this section, we analyze how genetic diversity recovers after a selective sweep in the data and in a mathematical model. (PDF) [file pgen.1004000.s008.pdf]

Supplementary Text S1

Loss and Recovery of Genetic Diversity in Adapting Populations of HIV
Pleuni S. Pennings , Sergey Kryazhimskiy , John Wakeley (PLoS Genetics)

Recovery of diversity

Because the patients were followed for approximately one year (median, appr. 200 generations [1]), it is possible to determine whether the viral populations in the patients recover from the observed selective sweep. We binned the observations in three bins. Bin 1: directly after the fixation event (the day the resistance mutation was detected, most likely this is shortly after it was fixed and allowed an increase in viral load). Bin 2: observations between 1 and 180 days after the fixation was observed. Bin 3: between 181 and 700 days after the fixation was observed. If a second drug resistance mutation became fixed in the virus of a patient the sample in which this was observed and any later samples were removed from the analysis.

In order to model the recovery of heterozygosity, we assume that during the periods between sweeps of resistance mutations there is no positive or balancing selection, only mutation, random drift, and negative selection. We further assume that negative selection on linked sites simply reduces the effective population size and may thus be captured by the random drift term in standard, single-locus population-genetic models [2].

We assume two alleles: the 'non-mutant' A_1 and the 'mutant' A_2, with relative fitnesses 1 and $1-s$, respectively. With probability u A_1 mutates to A_2, and with probability v A_2 mutates to A_1. Reproduction occurs according to the haploid Wright-Fisher model, with non-overlapping generations and constant population size N. If the current frequency of the mutant is x, then

$$x'' = \frac{x(1-s)(1-v) + (1-x)u}{1-sx} \tag{1}$$

gives the frequency after selection and mutation. The number of mutants in the next generation is binomial with parameters N and x'', so that its frequency X has expectation $E[X] = x''$ and variance $\mathrm{Var}[X] = x''(1-x'')/N$.

We are interested in the recovery of heterozygosity, and so consider

$$E[\Delta H] = E[2X(1-X) - 2x(1-x)] \tag{2}$$

$$= 2x''(1-x'')\left(1 - \frac{1}{N}\right) - 2x(1-x). \tag{3}$$

where the $(1 - \frac{1}{N})$ term reflects the loss of heterozygosity due to identity by descent (coalescence). We seek a simple heuristic formula that will aid in understanding the recovery of heterozygosity after a selective sweep. Assuming that s, u, v, and $1/N$ are all small, which is appropriate for HIV, and further

assuming that the mutant frequency x is small, gives

$$E[\Delta H] \approx 2u - \left(s + 3u + v + \frac{1}{N}\right) H, \tag{4}$$

in which $H = 2x(1-x) \approx 2x$. We apply this result to the recovery of heterozygosity at synonymous and non-synonymous sites heuristically using a continuous-time approximation.

For synonymous sites, we assume that all mutations are neutral and that $u, v \ll \frac{1}{N}$. Then $\frac{dH}{dt} = 2u - \frac{1}{N}H$, and if $H(0) = 0$, we have $H(t) = 2Nu(1 - \exp(-t/N))$. The response time, t_{half}, defined as the time it takes for H to recover 50% of its loss of diversity, can be found by solving $0.5 = \exp(-t_{half}/N)$ giving $t_{half} = N \log 2$. Note that under the assumption $u, v \ll \frac{1}{N}$, the response time is independent of the mutation rates and it is possible to estimate N independently from u. For non-synonymous sites, we assume that selection is stronger than both random drift and mutation, or and solve $\frac{dH}{dt} = 2u - sH$ to obtain $H(t) = 2(u/s)(1 - \exp(-st))$. In this case the response time is $t_{half} = s^{-1} \log 2$. Thus s may be estimated independently from u. These expressions for t_{half} illustrate that non-synonymous sites will recover faster than synonymous sites if $s > \frac{1}{N}$. Suppl Figure 2 shows that these approximate, heuristic expressions agree well with simulations as long as the mutation rate is sufficiently small.

Our conclusions are in agreement with previous results by Gordo and Disonisio [3] who described that more deleterious mutations approach mutation-selection equilibrium faster than less deleterious mutations. Song and Steinrücken [4] have recently described a method for studying the approach to stationarity of the entire distribution of allele frequencies, and also illustrated that recovery is faster when mutations are more deleterious.

References

1. Fu YX (2001) Estimating mutation rate and generation time from longitudinal samples of dna sequences. Molecular Biology and Evolution 18: 620-626.

2. Nagylaki T (1992) Introduction to Theoretical Population Genetics. Berlin-Heidelberg: Springer-Verlag.

3. Gordo I, Dionisio F (2005) Nonequilibrium model for estimating parameters of deleterious mutations. Physical Review E 71: 031907.

4. Song YS, Steinrücken M (2012) A simple method for finding explicit analytic transition densities of diffusion processes with general diploid selection. Genetics 190: 1117–1129.
